# Sensitivity of Various Indicators in a Mouse Sensitive Skin Model Treatment with 4-tert-Butylcyclohexanol and Pimecrolimus

**DOI:** 10.3390/ijms26094068

**Published:** 2025-04-25

**Authors:** Xueting Tang, Xueer Wang, Yarui Zhang, Qimei Chen, Shan Zhao, Xunhong Xu, Xinyu Yang, Xiaoran Liu, Lin Zhang, Min Zhang

**Affiliations:** 1 School of Public Health, Southern Medical University, Guangzhou 510515, China; tangxue7ing@163.com; 2 Department of Histology and Embryology, School of Basic Medical Sciences, Southern Medical University, Guangzhou 510515, China; wangxueer123@smu.edu.cn (X.W.);; 3 NMPA Key Laboratory for Safety Evaluation of Cosmetics, GDMPA Key Laboratory of Key Technologies for Cosmetics Safety and Efficacy Evaluation, Guangdong Provincial Key Laboratory of Construction and Detection in Tissue Engineering, Southern Medical University, Guangzhou 510515, China; 4 Guangzhou Dublin International College of Life Sciences and Technology, South China Agricultural University, Guangzhou 524100, China

**Keywords:** animal sensitive skin model, skin barrier, transient receptor potential vanilloid 1, 4-tert-butylcyclohexanol, pimecrolimus

## Abstract

The etiopathogenesis and treatment response of sensitive skin remain poorly understood. We used 4-tert-butylcyclohexanol (4-TBLH) and 1% pimecrolimus ointment to treat sensitive skin in mice models constructed using tape stripping, propylene glycol, and capsaicin. This study aimed to further investigate the sensitivity and responsiveness of this sensitive mouse skin model. Sensitivity and responsiveness were assessed by measuring transepidermal water loss (TEWL), skin hydration, skin flakes, vascular dilatation, itching, stinging, and histological changes, including mast cell, lymphocyte, and granulocyte infiltration, tumor necrosis factor-α (TNF-α) expression, and transient receptor potential vanilloid 1 receptor (TRPV1) expression. The application of 4-TBLH and pimecrolimus revealed distinct responses in skin sensitivity indicators, including TEWL, capillary dilation, and mass cell activity, depending on the treatment timing and substance used. The prophylactic and therapeutic applications of 4-TBLH revealed distinct responses in skin sensitivity indicators, including skin flakes, TEWL, itching, stinging, epidermal thickness, mast cell activity, TNF-α, and TRPV1 expression. The prophylactic and therapeutic applications of pimecrolimus ointment revealed distinct responses in skin sensitivity indicators, including skin flakes, skin water content, itching, epidermal thickness, mast cell activity, CD45, CD11b, TNF-α, and TRPV1 expression. The mouse sensitive skin model demonstrates robust sensitivity and responsiveness to different treatment factors, and the model can be applied to the development of prophylactic and therapeutic medications for sensitive skin.

## 1. Introduction

Sensitive skin arises from a complex interplay of skin barrier dysfunction, neurovascular mechanisms, and immune-mediated inflammation [1,2]. Among these, impaired skin barrier function represents a critical and central factor in the development of sensitive skin [3,4]. Physiological assessments of patients with sensitive skin have demonstrated increased transepidermal water loss (TEWL) and reduced stratum corneum moisture content, indicative of compromised skin barrier integrity [5].

Skin barrier destruction not only allows environmental stimuli to directly activate the transient receptor potential vanilloid 1 (TRPV1) receptor but also diminishes the protective functions of nerve endings. The upregulation of TRPV1 can lead to a significant increase in sensory nerve signaling, which mediates symptoms such as skin pain, itching, and burning sensations [6,7,8,9]. Notably, TRPV1 is expressed not only in sensory nerve endings but also in keratinocytes and mast cells. Its activation could stimulate keratinocytes to release inflammatory mediators, such as tumor necrosis factor-alpha (TNF-α) and interleukin-6 (IL-6), and promote the production of vascular endothelial growth factor (VEGF), contributing to vascular reactivity, vasodilatation, and increased vascular density in the superficial skin layers [10,11]. Moreover, TRPV1 activation and the chemotaxis of inflammatory factors promote mast cell recruitment and degranulation, exacerbating local immune and inflammatory responses. This process further manifests as erythema, a burning sensation, and itching [12,13,14].

In our previous study, we constructed a mouse model for sensitive skin by first employing tape stripping to disrupt the skin barrier, then applying propylene glycol to alter the lipid composition between skin keratinocytes, and finally applying capsaicin to activate TRPV1 receptors [15]. This model not only reproduced the typical neurogenic symptoms mediated by TRPV1 upregulation (including itchiness, tingling, and capillary vasodilation) but also activated keratinocytes and mast cells through TRPV1 to trigger an inflammatory response, which was specifically manifested as significantly increased TRPV1 expression accompanied by TRPV1-dependent TNF-α release, leading to increased vasodilation and capillary perfusion. TRPV1 mediated neuropeptide release promotes mast cell degranulation and forms a neuro-immune positive feedback loop, which ultimately lead to the simultaneous appearance of sensitive skin phenotypes, such as skin flushing, increased vascular reactivity, impaired barrier function (increased TEWL value, decreased skin water content), and characteristic immune cell (mast cell) infiltration.

We hypothesized that this model could be used for studying the pathogenesis of sensitive skin and assessing whether cosmetic ingredients and drugs could be used to prevent and treat sensitive skin. Based on this hypothesis, we selected a cosmetic ingredient, 4-TBLH, and a therapeutic drug, 1% pimecrolimus ointment, for treating sensitive skin. 4-TBLH, an ingredient commonly added to various cosmetic products for sensitive skin, is a TRPV-1 antagonist that reduces a series of reactions caused by TRPV1 activation [16,17], and 1% pimecrolimus ointment, a widely used medication for treating sensitive skin, is a calmodulin phosphatase inhibitor that inhibits T-cell activation and cytokine production, regulating the sensory nerve fiber activity and reducing the release of inflammation-associated factors like interleukins to alleviate itchiness, burning sensations, and skin inflammation [18,19]. In this study, we aimed to assess the changes in skin sensitivity parameters after the prophylactic and therapeutic application of 4-TBLH and pimecrolimus. For this, we used 4-TBLH and pimecrolimus ointment on the mice’s skin immediately after creating the sensitive skin models to explore their preventive effects on the occurrence of sensitive skin. Simultaneously, 4-TBLH and 1% pimecrolimus ointment were applied after a few days to investigate their therapeutic effect on sensitive skin in mice with significant skin symptoms. We expect that after the prophylactic and therapeutic application of this sensitive mouse model to 4-TBLH, the changes in various indices will be different, and the application of pimecrolimus will also show similar changes. After comparing 4-TBLH and pimecrolimus for prophylactic application or therapeutic application, the changes in various indices were also different.

## 2. Results

### 2.1. External Manifestations of Skin After 4-TBLH and Pimecrolimus Treatments in a Mouse Sensitive Skin Model

**Figure 1:**
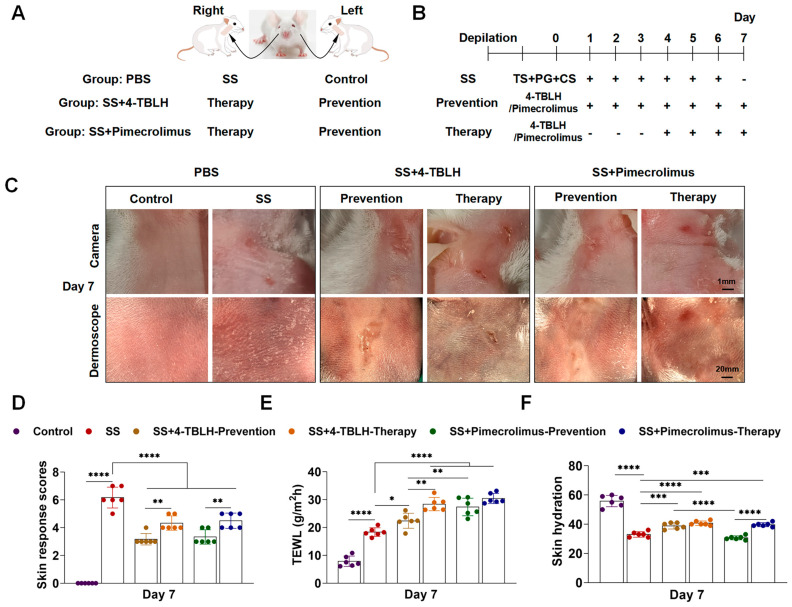
External manifestations of sensitive skin in mice after prophylactic and therapeutic application of 4-TBLH and pimecrolimus with changes in skin response scores, TEWL values, and skin hydration. (**A**). Grouping of KM mice for sensitive skin modeling and treatment applied to the right and left cheeks. (**B**). Methods for creating the sensitive skin model in KM mice, along with prophylactic and therapeutic applications of 4-TBLH and pimecrolimus, +: corresponding treatment applied; -: corresponding treatment not applied. (**C**). Camera and dermatoscopic photographs of the mice showing skin appearance on day 7 in each group. (**D**). Skin response scores and statistical analysis between groups. (**E**). TEWL values and statistical analysis across groups. (**F**). Skin hydration values and statistical analysis across groups. * *p* < 0.05; ** *p* < 0.01; *** *p* < 0.001; **** *p* < 0.0001; (*n* = 6), Abbreviations: KM, Kunming; SS, sensitive skin; 4-TBLH, 4-tert-butylcyclohexanol; TS, tape stripping; PG, 1,2- propylene glycol; CS, capsaicin. TEWL, transepidermal water loss.

In the mouse sensitive skin model, external skin changes were observed following 7 days of prophylactic application and 4 days of therapeutic application of 4-TBLH and 1% pimecrolimus ointment to the sensitive skin of the mice (Figure 1B). Changes in skin appearance in the different treatment groups were recorded using a digital camera and dermatoscope, and skin reactions (erythema, edema formation, and skin desquamation) were scored according to the method described by Nisbet based on an adapted version of the Draize and Kligman scale [20,21]. The overall intensity of the reaction was categorized based on the score. A total score of 0–2 was classified as no reaction, 3–4 as a mild reaction, 5–8 as a moderate reaction, and >8 as a strong reaction.

On day 7, the control group had a normal skin appearance and wrinkles. In contrast, the sensitive skin groups had red scaly skin, localized lesions, and a stronger skin reaction, similar to the severe manifestations of atopic skin [22,23], compared to the control group (*p* < 0.0001). After prophylactic and therapeutic applications to the 4-TBLH group, redness, localized lesions, and desquamation persisted, with the whole skin exhibiting mild reactions. Notably, prophylactic treatment reduced flaking compared to therapeutic treatment (*p* = 0.0053). Similarly, redness, lesions, and flaking were observed in the pimecrolimus-treated group, with the whole skin exhibiting mild reactions. However, prophylactic treatment resulted in less scaling than the therapeutic treatment (*p* = 0.0053) (Figure 1C,D and Table 1).

### 2.2. Changes in Skin Barrier Function and Epidermal Moisture Levels After 4-TBLH and Pimecrolimus Treatment in a Mouse Sensitive Skin Model

Changes in TEWL and epidermal moisture content reflect skin barrier function [24,25,26]. After prophylactic and therapeutic application of 4-TBLH and pimecrolimus during the modeling of mouse sensitive skin, TEWL was measured using a transdermal water loss instrument (AF200). On day 7, the TEWL value in the mouse-sensitive skin model group was significantly higher than that in the control group, increasing to (18.35 ± 1.55) g/m^2^h (*p* < 0.0001). After 7 days of prophylactic application, the TEWL values in the 4-TBLH group increased to (22.42 ± 2.67) g/m^2^h, which was higher than those in the simple sensitive skin model group (*p* = 0.0490). In contrast, 4 days of therapeutic application of 4-TBLH significantly elevated the TEWL values to (28.40 ± 2.37) g/m^2^h, exceeding those observed in the simple model group (*p* < 0.0001). Moreover, the TEWL values in the therapeutic application group were significantly higher than those in the prophylactic application group (*p* = 0.0013).

Similarly, in the pimecrolimus group, TEWL values of the sensitive skin increased after 7 days of prophylactic application and 4 days of therapeutic application, both exceeding the values of the simple sensitive skin model group (*p* < 0.0001 for all). However, the TEWL in the therapeutic application group was higher than that in the prophylactic application group, although this difference was not statistically significant. Comparing the prophylactic effects of 4-TBLH and pimecrolimus, the TEWL values in the pimecrolimus group were significantly higher than those in the 4-TBLH group (*p* = 0.0100) (Figure 1E, Table 2).

The skin moisture content in each group was assessed using the Skin Multi-Channel Physiology Instrument (DUB-MSTC). In the simple sensitive skin model group, the skin moisture content was significantly reduced to (33.00 ± 1.90) compared to that in the control group (55.83 ± 3.87) (*p* < 0.0001). The prophylactic application of pimecrolimus slightly decreased the skin moisture content compared to the simple sensitive skin model group but showed no significant difference. In the other groups treated with 4-TBLH and pimecrolimus, the moisture content ranged from (38.83 ± 2.14) to (40.67 ± 1.51), slightly higher than that in the simple sensitive skin group (*p* = 0.0010, *p* < 0.0001, *p* = 0.0002, respectively). Within the pimecrolimus group, therapeutic application resulted in higher skin water content compared to that following prophylactic application (*p* < 0.0001). Comparing the prophylactic treatments, the skin water content in the 4-TBLH group was significantly higher than that in the pimecrolimus group (*p* < 0.0001) (Figure 1F, Table 2).

### 2.3. Changes in Capillary Dilation and Perfusion Volume After Treatment with 4-TBLH and Pimecrolimus in a Mouse Sensitive Skin Model

**Figure 2:**
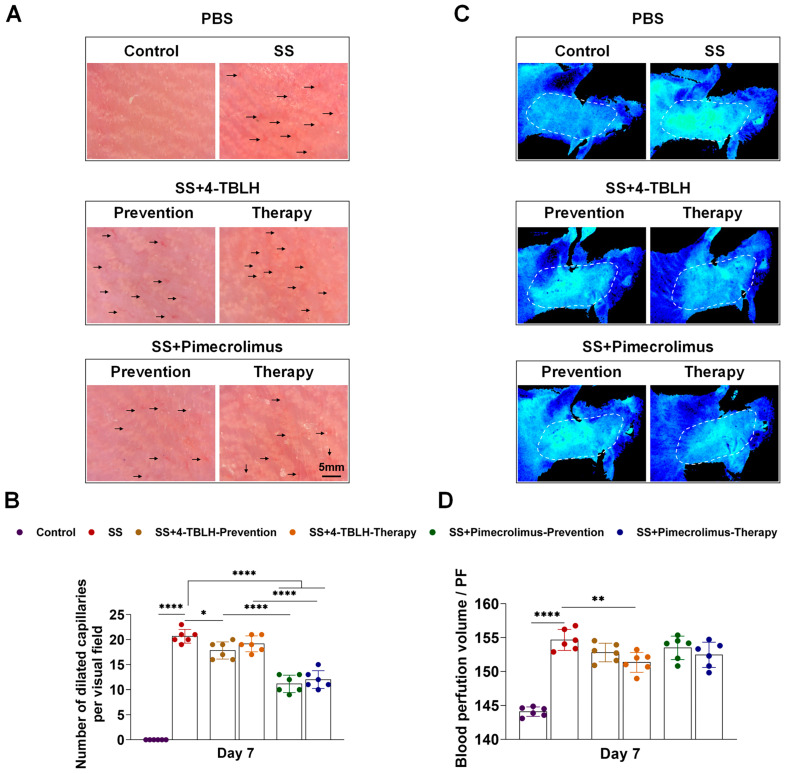
Changes in capillary dilation and blood flow indices in sensitive skin of mice after prophylactic versus therapeutic application of 4-TBLH and pimecrolimus. (**A**). High magnification gross microscopy images showing capillary dilatation in each group, scale bar: 5 mm. (**B**). Statistical analysis of the number of dilated capillaries per unit area in each group. (**C**). Representative infrared blood flow images showing local average blood perfusion in each group (additional images for all mice are provided in Supplementary Figure S1). (**D**). Statistical analysis of the local average blood perfusion of the skin within the unit area of each group. * *p* < 0.05; ** *p* < 0.01; **** *p* < 0.0001; (*n* = 6). Abbreviations: SS, sensitive skin; 4-TBLH, 4-tert-butylcyclohexanol.

After prophylactic and therapeutic application of 4-TBLH and pimecrolimus during sensitive skin modeling in mice, skin capillary dilatation was observed on day 7 using high magnification hair microscopy (Figure 2A,B, Table 2). No capillaries were visible in the normal skin of the control group, whereas significantly more dilated capillaries were observed in the sensitive skin model group. The number of dilated capillaries per unit area (20.67 ± 1.37) was significantly higher in the sensitive skin model group compared to the control group (*p* < 0.0001), resembling capillary dilatation observed in individuals with sensitive skin [27,28]. Prophylactic application of 4-TBLH reduced the localized capillary count (17.83 ± 1.72) (*p* = 0.0298), whereas therapeutic application showed a less pronounced reduction (19.17 ± 1.62), with no significant difference compared to that in the sensitive skin model group alone. Conversely, pimecrolimus significantly reduced capillary dilation in prophylactic (11.17 ± 1.72) and therapeutic (12.00 ± 1.79) applications, showing significant differences compared to the sensitive skin model group (*p* < 0.0001 for all). When comparing the prophylactic or therapeutic applications of both treatments, pimecrolimus yielded a significantly lower number of dilated capillaries per unit area than the corresponding prophylactic and therapeutic applications of 4-TBLH (*p* < 0.0001 for all).

Infrared blood flow imaging revealed that the localized mean blood perfusion in the skin of the sensitive skin model group was significantly elevated to (154.70 ± 1.55), compared to the normal skin of the control group (144.10 ± 0.69) (*p* < 0.0001). Following 4-TBLH and pimecrolimus application, prophylactic and therapeutic applications reduced localized mean blood perfusion compared to the sensitive skin model group, ranging from (151.30 ± 1.47) to (153.50 ± 1.73). However, only the therapeutic application of 4-TBLH resulted in a significant reduction compared to the sensitive skin model group (*p* = 0.0071) (Figure 2C,D, Table 2). Representative images are shown in Figure 2C, with complete imaging data for all mice provided in Supplementary Figure S1.

### 2.4. Changes in Itching and Tingling Sensation of the Skin After Treatment with 4-TBLH and Pimecrolimus in a Mouse Model of Sensitive Skin

Each group of mice underwent a 45-min behavioral video recording, after which scratching (using the ipsilateral hind limb) and cheek wiping (using the ipsilateral forelimb) behaviors were observed, counted, and statistically analyzed. Regarding this behavior, there was no significant difference between the control group on day D0 and the control group on day D7, whereas a significant increase in the number of scratches and wipes was observed in the sensitive skin groups on day D7 (*p* < 0.0001). This implies that changes in the number of scratches and wipes in each of the 4-TBLH and pimecrolimus groups at day D7 were related to the application of the corresponding treatment in each group.

In the control group, the number of scratching and wiping behaviors of mice after depilatory cream that was used to remove hair from the cheeks to the forelimbs was 27.83 ± 2.56 and 21.83 ± 2.93, respectively. In contrast, mice in the sensitive skin model exhibited significantly more scratching and wiping behaviors ([81.67 ± 3.93] and [116.30 ± 5.20], respectively), indicating a heightened sensation of itching and tingling (*p* < 0.0001 for all).

Prophylactic and therapeutic applications of 4-TBLH significantly reduced scratching ([49.00 ± 2.68] [*p* < 0.0001] and [56.83 ± 2.64] [*p* < 0.0001], respectively) and wiping behaviors ([67.50 ± 5.13] [*p* < 0.0001] and [59.67 ± 5.75] [*p* < 0.0001], respectively), compared to those in the sensitive skin model alone. However, the values remained higher than those in the control group. Similarly, prophylactic and therapeutic applications of pimecrolimus significantly reduced scratching ([28.17 ± 2.48] [*p* < 0.0001] and [13.17 ± 1.94] [*p* < 0.0001], respectively) and rubbing behaviors ([26.33 ± 2.25] [*p* < 0.0001] and [25.00 ± 1.87] [*p* < 0.0001], respectively), showing a significant difference from the sensitive skin model group, with values higher than those of the control group; however, this difference was not statistically significant.

**Figure 3:**
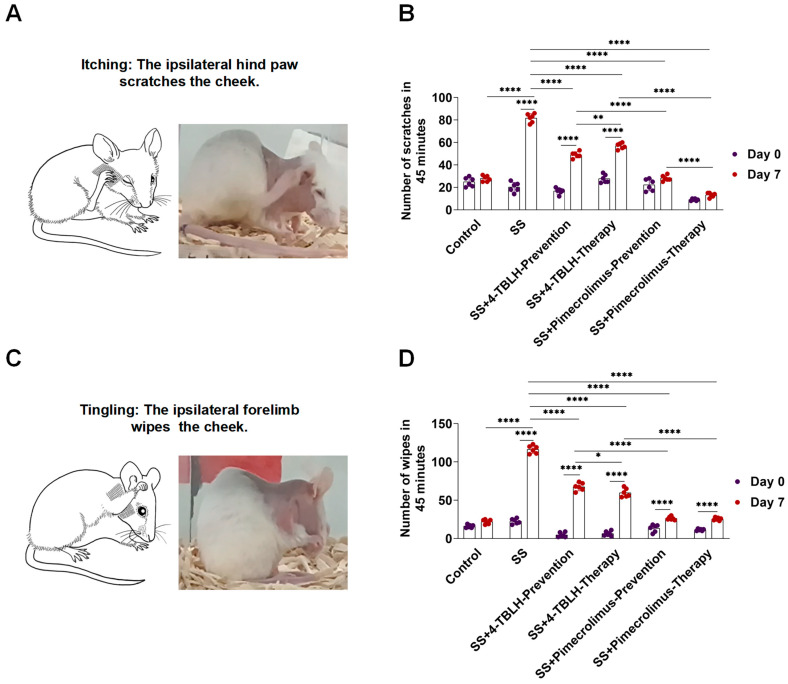
Changes in sensitized skin itching and stinging in mice after prophylactic and therapeutic application of 4-TBLH and pimecrolimus. (**A**). Pattern diagram and legend of mouse scratching. (**B**). Number of scratches in each group at day 0 and day 7 and statistical analysis. (**C**). Pattern diagram and legend of mice wiping. (**D**). Number of wipes in each group at day 0 and day 7 and statistical analysis. * *p* < 0.05; ** *p* < 0.01; **** *p* < 0.0001; (*n* = 6). Abbreviations: SS, sensitive skin; 4-TBLH, 4-tert-butylcyclohexanol.

Within the 4-TBLH group, prophylactic application resulted in fewer scratching behaviors but more wiping behaviors compared to those following the therapeutic application (*p* = 0.0075, *p* = 0.0226, respectively). Conversely, in the pimecrolimus group, therapeutic application resulted in fewer scratching behaviors compared to those following prophylactic application (*p* < 0.0001). When comparing 4-TBLH and pimecrolimus, prophylactic and therapeutic applications of pimecrolimus resulted in fewer scratching and wiping behaviors than those following 4-TBLH application (*p* < 0.0001 for all) (Figure 3, Table 2).

### 2.5. Lactic Acid Stimulation Test in a Sensitive Skin Model of Mice Treated with 4-TBLH and Pimecrolimus

Lactic acid stimulation is a semi-subjective evaluation method for evaluating skin sensitivity, as reported in the literature [15]. A 0.5% lactic acid aqueous solution was applied to test sites on the skin of each group of mice to assess skin sensitivity. Behavioral responses, including scratching and wiping, were recorded during a 45-min video and analyzed before and after the lactic acid stimulation.

**Figure 4:**
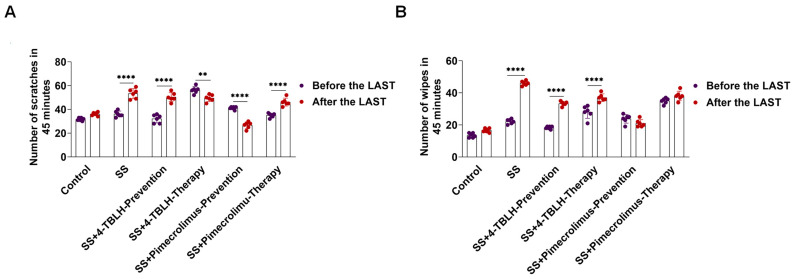
Reactivity of sensitive mice skin to the lactic acid test after prophylactic and therapeutic application of 4-TBLH and pimecrolimus. (**A**). Statistical analysis of scratching behaviors before and after lactic acid stimulation in each group. (**B**). Statistical analysis of wiping behaviors before and after lactic acid stimulation in each group. ** *p* < 0.001; **** *p* < 0.0001; (*n* = 6). Abbreviations: SS, sensitive skin; 4-TBLH, 4-tert-butylcyclohexanol.

As shown in Figure 4 and Table 3, the application of a 0.5% lactic acid aqueous solution on day 7 to the skin of control mice resulted in no significant changes in scratching and wiping behaviors before and after application, suggesting that the solution did not irritate the skin of normal mice. In the sensitive skin model, application of a 0.5% lactic acid aqueous solution significantly increased scratching (53.33 ± 4.23) and wiping behaviors (46.00 ± 1.41) compared to the baseline of scratching (36.17 ± 2.48) and wiping behaviors (22.17 ± 1.47) (*p* < 0.0001 for all). This suggests that the skin at the modeling site was in a sensitive state.

In the 4-TBLH prophylactic application group, scratching and wiping behaviors significantly increased after lactic acid stimulation compared to those at baseline (*p* < 0.0001 for all). Similarly, wiping behaviors were significantly elevated in the 4-TBLH therapeutic application group after stimulation (*p* < 0.0001). These findings suggest that prophylactic 4-TBLH application does not enhance the tolerance of sensitized skin. In the pimecrolimus prophylactic application group, scratching and wiping behaviors did not significantly increase after lactic acid stimulation. In the pimecrolimus therapeutic application group, scratching behaviors were elevated (*p* < 0.0001), whereas wiping behaviors showed no significant change. These results suggest that prophylactic pimecrolimus application enhances the tolerability of sensitive skin.

### 2.6. Changes in Skin Histologic Levels After Treatment of 4-TBLH and Pimecrolimus in a Mouse Model of Sensitive Skin

**Figure 5:**
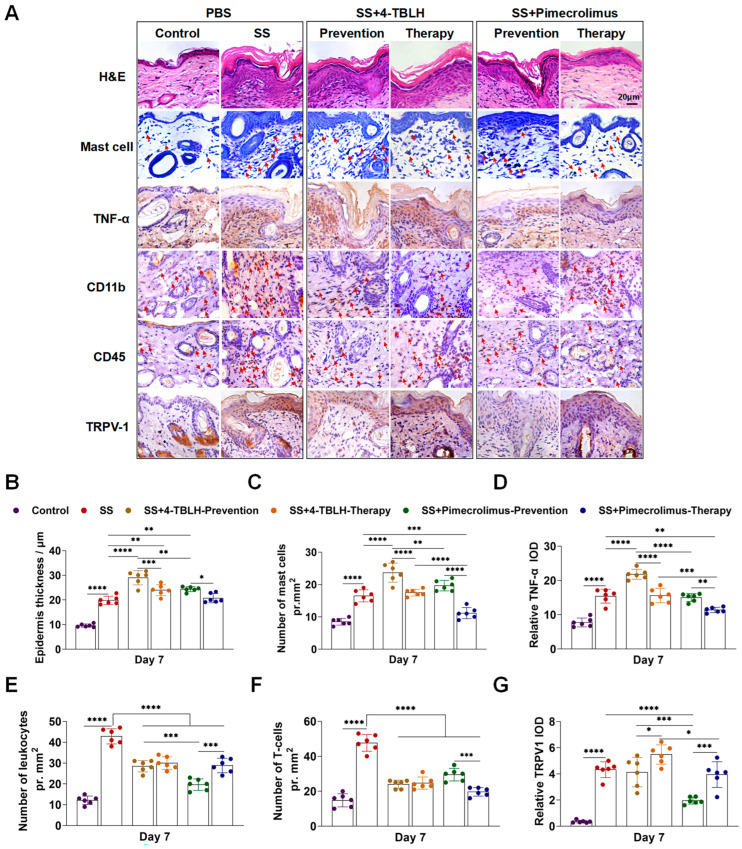
Histologic changes and statistical analysis of mouse sensitive skin model after prophylactic versus therapeutic application of 4-TBLH and pimecrolimus. (**A**). Representative images of skin sections stained with hematoxylin and eosin (H&E) for general morphology, toluidine blue staining for mast cells, and immunohistochemistry for TNF-α, CD11b, CD45, and TRPV1 expression. (**B**). Statistical analysis of epidermal thickness in each group. (**C**). Statistical analysis of mast cell count in each group. (**D**). Statistical analysis of the optical density value of TNF-α per unit area in each group. (**E**). Statistical analysis of CD11b-positive cell count per unit area in each group. (**F**). Statistical analysis of CD45-positive cell count per unit area in each group. (**G**). Statistical analysis of the optical density value of TRPV1 per unit area in each group. * *p* < 0.05; ** *p* < 0.01; *** *p* < 0.001; **** *p* < 0.0001; (*n* = 6). Abbreviations: SS, sensitive skin; 4-TBLH, 4-tert-butylcyclohexanol.

On day 7, paraffin sections of skin tissues from each group were subjected to H&E, toluidine blue, and immunohistochemical staining for TNF-α, CD11b, CD45, and TRPV1. These methods were used to evaluate molecular changes in sensitive skin tissues following prophylactic and therapeutic applications of 4-TBLH and pimecrolimus (Figure 5). H&E staining revealed that the epidermis of normal mouse skin had only 2–3 cell layers and an average thickness of (9.62 ± 0.65) μm. In contrast, the epidermis in the sensitive skin model group thickened significantly to (19.85 ± 1.68) μm, with distinct layers of stratum basale, spinosum, granulosum, and corneum clearly visible. Statistical analysis confirmed the significant difference in epidermal thickness (*p* < 0.0001) (Figure 5A,B, and Table 4).

In the 4-TBLH group, the epidermal thickness in the sensitive skin model group increased after prophylactic (29.02 ± 2.89 μm) and therapeutic applications (24.00 ± 2.22 μm) (*p* < 0.0001, *p* = 0.0069, respectively), with therapeutic application resulting in a lower thickness than prophylactic application (*p* = 0.0008). In the pimecrolimus group, prophylactic application resulted in a significantly greater epidermal thickness compared to the sensitive skin group (*p* = 0.0027). However, therapeutic application resulted in a significantly lower thickness, showing no significant difference from the epidermal thickening in the sensitive skin group alone.

Toluidine blue staining was performed to observe mast cells (Figure 5A,C, and Table 4). The results showed a significant increase in mast cell numbers in the simple mouse-sensitive model skin group compared to that in the control group (*p* < 0.0001). After 4-TBLH prophylactic application, the number of mast cells increased significantly (*p* < 0.0001). In contrast, the therapeutic application of 4-TBLH resulted in a mass cell count comparable to that in the sensitive skin model group but significantly lower than that in the prophylactic application group (*p* < 0.0001).

In the pimecrolimus group, prophylactic application did not significantly alter the number of mast cells compared to that in the sensitive skin model. However, the therapeutic application of pimecrolimus significantly reduced the number of mast cells compared to that in the sensitive skin model and pimecrolimus prophylactic application groups (*p* = 0.0003, *p* < 0.0001, respectively). Although the number of mast cells in the therapeutic pimecrolimus group remained higher than that in the control group, the difference was not statistically significant. Furthermore, mast cell counts in prophylactic and therapeutic pimecrolimus application groups were significantly lower than those in the corresponding 4-TBLH groups (*p* = 0.0080, *p* < 0.0001, respectively).

The results of TNF-α immunohistochemistry (Figure 5A,D, and Table 4) revealed a significant increase in TNF-α expression levels in the sensitive skin group compared to the control group (*p* < 0.0001). Following the prophylactic application of 4-TBLH, TNF-α expression levels in the sensitive skin tissues were significantly higher than those of the sensitive skin modeling group (*p* < 0.0001). However, after the therapeutic application of 4-TBLH, TNF-α expression levels were not significantly different from those observed in the simple sensitive skin group. In the pimecrolimus group, prophylactic application did not result in a significant change in TNF-α expression in the sensitive skin tissues compared to the sensitive skin modeling group. In contrast, therapeutic application significantly reduced TNF-α expression compared to the sensitive skin model group (*p* = 0.0011). Furthermore, TNF-α expression levels in the prophylactic and therapeutic pimecrolimus application groups were significantly lower than those in the corresponding 4-TBLH groups (*p* < 0.0001, *p* = 0.0006, respectively).

Immunohistochemical detection of CD11b-positive leukocytes, including granulocytes and macrophages, revealed a significant increase in CD11b-positive cells in the sensitive skin group compared to the control group (*p* < 0.0001) (Figure 5A,E, and Table 4). Moreover, prophylactic and therapeutic applications of 4-TBLH and pimecrolimus significantly reduced the number of CD11b-positive cells in sensitive skin tissues compared to those in the sensitive skin model group (*p* < 0.0001 for all). Notably, the number of CD11b-positive cells in the pimecrolimus prophylactic application group was significantly lower than in the 4-TBLH prophylactic application group and the pimecrolimus therapeutic application group (*p* = 0.0002, *p* = 0.0001, respectively).

CD45 immunohistochemical staining for T-lymphocytes demonstrated a significant increase in CD45-positive cells in the sensitive skin model group compared to that in the control group (*p* < 0.0001) (Figure 5A,F, and Table 4). Additionally, prophylactic and therapeutic applications of 4-TBLH and pimecrolimus significantly decreased the number of CD45-positive cells in the sensitive skin tissues relative to those in the sensitive skin model group (*p* < 0.0001 for all). Furthermore, the therapeutic application of pimecrolimus resulted in significantly fewer CD45-positive cells compared with its prophylactic application group (*p* = 0.0005).

Immunohistochemical detection of TRPV1 expression revealed a significant increase in TRPV1 expression levels in the sensitive skin tissues of mice compared to those in the control group (*p* < 0.0001) (Figure 5A,G, and Table 4). However, there was no significant difference in TRPV1 expression levels between the sensitive skin tissue group and the 4-TBLH prophylactic application group. The TRPV1 expression level in the skin of the 4-TBLH therapy group was increased, but did not show a significant difference from that of the sensitive skin alone group. In contrast, pimecrolimus prophylactic application resulted in significantly lower TRPV1 expression levels compared to those in the sensitive skin model group (*p* < 0.0001). Furthermore, there was no significant difference in TRPV1 expression between the pimecrolimus therapeutic application group and the sensitive skin model group. The pimecrolimus prophylactic and therapeutic application groups exhibited significantly lower TRPV1 expression than their corresponding 4-TBLH groups (*p* = 0.0002, *p* = 0.0120, respectively).

## 3. Discussion

In our previous study, we constructed a mouse model of sensitive skin using tape stripping, propylene glycol, and capsaicin [15]. This model demonstrated several characteristics typical of sensitive skin, including increased skin scales, elevated TEWL, reduced water content in the stratum corneum, local vasodilatation, and increased local blood flow. Behavioral tests revealed an increase in scratching and wiping behaviors. Histological investigations showed increased epidermal thickness, a higher number of mast cells, and elevated granulocyte and lymphocyte counts. At the molecular level, the typical expression of TRPV1 and TNF-α was elevated. Based on these findings, we concluded that this mouse-sensitive skin model is a dry secondary highly sensitive skin model, according to symptom severity [16,29,30] and Baumann’s skin typing system [31].

In order to further verify the sensitivity and responsiveness of various indicators in this mouse sensitive skin model to treatment, we selected 4-TBLH, an ingredient commonly added to various cosmetic products for sensitive skin, and 1% pimecrolimus ointment, a widely used medication for treating sensitive skin. We observed changes in multiple indicators following these treatments. 4-TBLH is a TRPV1 antagonist that reduces a series of reactions caused by TRPV1 activation [16,17]. Pimecrolimus ointment is a calmodulin phosphatase inhibitor that inhibits T-cell activation and cytokine production, regulating the sensory nerve fiber activity and reducing the release of inflammation-associated factors like interleukins to alleviate itchiness, burning sensation, and skin inflammation [18,19].

To observe changes in various indices between the same treatment factors but with different treatment times, we divided the two treatment factors into prophylactic and therapeutic application groups. The prophylactic application was administered throughout the entire period of mouse-sensitized skin modeling. In contrast, the therapeutic application was administered for 4 days after 3 days of modeling, when the skin of the mice showed clear signs of sensitivity. Relative to untreated mouse-sensitive skin, the prophylactic application of 4-TBLH, elevated TEWL, increased water content, decreased the number of dilated capillaries, showed no significant change in mean blood perfusion, reduced the number of scratches and wipes, increased the epidermal thickness, increased the number of mast cells, elevated TNF-α expression, and decreased the numbers of CD45+ and CD11b+ cells. However, the expression of TRPV1 did not change significantly. After therapeutic application of 4-TBLH, TEWL was further elevated, water content increased, there was no change in the number of dilated capillaries, mean blood perfusion decreased, the number of scratches and wipes decreased, epidermal thickness increased, there was no significant change in the number of mast cells, no change in the expression of TNF-α, a decrease in the numbers of CD45+ and CD11b+ cells, and TRPV1 expressed was no significant change.

Comparison between prophylactic and therapeutic applications of 4-TBLH revealed significant differences in TEWL, the number of scratches and wipes, skin epidermal thickness, mast cell count, TNF-α expression, and TRPV1 expression. These results suggest that this mouse model of sensitized skin has better sensitivity and responsiveness to the same substance with different treatment times. It is also suggested that prophylactic application of 4-TBLH alone may not be effective under the hypersensitive state of the disrupted skin barrier, likely due to the limitation of single-factor antagonism on TRPV1. The combination of prophylactic and therapeutic treatments may provide a more effective alleviating response.

Compared to untreated sensitive skin in mice, pimecrolimus prophylactic application resulted in elevated TEWL, no significant change in water content, a decreased number of dilated capillaries, and no significant change in mean blood perfusion. It reduced the number of scratches and wipes and increased the epidermal thickness and mast cell count but did not significantly affect TNF-α expression. It also decreased the CD45+ and CD11b+ cell count and significantly reduced TRPV1 expression. In contrast, pimecrolimus therapeutic application resulted in elevated TEWL, increased water content, a reduced number of dilated capillaries, and fewer scratches and wipes. It caused no significant change in mean blood perfusion or epidermal thickness but significantly reduced the mast cell count and TNF-α expression. It also decreased the CD45+ and CD11b+ cell count, but TRPV1 expression remained unchanged.

The comparison of prophylactic and therapeutic applications of pimecrolimus revealed significant differences in the skin water content, number of scratches, epidermal thickness, mast cell count, TNF-α expression, and TRPV1 expression, suggesting that the sensitive skin model exhibited higher sensitivity and responsiveness to the same substance at different treatment times. When comparing the prophylactic application of 4-TBLH and pimecrolimus, significant differences were observed in the TEWL, skin water content, number of dilated capillaries, scratches and wipes, epidermal thickness, number of mast cells, TNF-α expression, and TRPV1 expression. Similarly, significant differences between the two substances during therapeutic application were noted in the number of dilated capillaries, scratches and wipes, mast cell count, TNF-α expression, and TRPV1 expression. These findings highlight that the sensitivity and responsiveness of the model to treatment varied depending on the substance applied.

In the lactic acid stimulation test used to assess skin sensitivity in each group of mice, the skin of mice treated with 4-TBLH prophylactically and therapeutically exhibited significant reactivity to lactic acid stimulation. However, the degree of reactivity differed between the two treatment regimens. In contrast, mice treated with pimecrolimus prophylactically and therapeutically showed no significant skin reactivity following lactic acid stimulation, suggesting that pimecrolimus has an alleviating effect on skin sensitivity in this mouse sensitivity model.

Notably, recent studies have highlighted the pivotal role of cutaneous neuroendocrine signaling and the epidermal neuroregulatory network in analogous physiological and pathological processes. Clinical manifestations of sensitive skin-including erythema, vasodilation, and barrier dysfunction may be influenced by neuroendocrine–immune interactions and epidermal neural modulation. Future studies could validate this hypothesis by detecting relevant neuropeptides [32,33,34].

In summary, this mouse sensitive skin model demonstrated robust sensitivity and responsiveness to various parameters, including TEWL, skin water content, vasodilatation, itching sensation and tingling sensations, mast cell count, TNF-α expression, CD45+ and CD11b+ cell count, and TRPV1 expression, when exposed to different substances and treatment durations. This highlights its potential as an acute sensitization model for evaluating the preventive and therapeutic efficacy of various substances in mitigating skin sensitivity. Looking ahead, this sensitive skin model has broad application prospects, including testing and evaluating the effectiveness of various new drugs or treatments for sensitive skin. Additionally, incorporating other factors during the modeling process could help design responses tailored to multiple skin sensitivity indicators, enabling a more comprehensive study of the complex interactions among neural, immune, and skin systems in the pathogenesis of sensitive skin. Although this model is valuable for acute sensitive skin model sensitization research, its current limitations include the rapid recovery of skin indicators within 2 days after modeling cessation, therefore making it unsuitable for studying post-modeling treatments. Future developments should focus on creating chronic or diverse types of sensitive skin models, further advancing research, and benefiting the scientific community.

## 4. Materials and Methods

### 4.1. Experimental Animals

Owing to the lack of active melanocytes in the skin and hair follicles of KM mice, their skin color does not change with the hair follicle cycle. Moreover, they have a stable skin phenotype, which can be visualized easily; therefore, they are suitable for the construction of sensitive skin mouse models [35,36].

Eighteen female SPF grade KM mice aged 6–8 weeks were obtained from the Experimental Animal Center of Southern Medical University [SYXK (Guangdong) 2021-0041] and raised in the Animal Laboratory Department of Southern Medical University [SYXK (Guangdong) 2021-0167]. The mice were maintained under controlled environmental conditions: a temperature regimen of 24 °C ± 2 °C, relative humidity of 50 ± 10%, and a 12-h light/dark cycle. Each mouse was individually housed in a plastic feeding cage with unimpeded access to standard animal feed and water. All experimental methods and procedures adhered to the Guide for Animal Experimentation and were approved by the Experimental Animal Ethics Committee of Southern Medical University (experimental protocol code: SMUL202309002; approval date: 9 September 2023).

### 4.2. Main Reagents and Instruments

The study utilized a variety of reagents and equipment. The 1% pimecrolimus ointment was purchased from MEDA Manufacturing, Merignac, France; 4-TBLH was procured from Shanghai Maclin Biochemical Technology Co., Ltd., in Shanghai, China (10 mg of 4-TBLH powder dissolved in 1 mL PBS, configured in a 1% 4-TBLH solution); 1,2-propylene glycol (PG) and capsaicin (CS) (Solarbio) were bought from Guangzhou Saiguo Biotechnology Co., Ltd., Guangzhou, China; Lactic acid (Meryer) and purchased from Shanghai Mindray Biochemical Technology Co., Ltd., Shanghai, China; Toluidine blue and hematoxylin and eosin (H&E) were acquired from Fuzhou Maixin Biotechnology Development Co., Ltd., within Fuzhou, China. For immunohistochemistry, TNF-α (Proteintech (Rosemont, IL, USA), Cat no. 17590-1-AP), CD45 (Proteintech (Rosemont, IL, USA), Cat no. 20103-1-AP), TRPV1 (abcam (Cambridge, UK), Cat. no. Ab305178), and cluster of differentiation 11b (CD11b) antibodies (abcam (Cambridge, UK), Cat no. 133357) were acquired from Guangzhou Chuangwei Biotechnology Co., Ltd., Guangzhou, China. The goat anti-rabbit and goat anti-mouse immunoglobulin G polymer conjugated with enzymes, along with 3,3′-diaminobenzidine (DAB) reagent (Zhongshan Jinqiao), were purchased from Guangzhou Demeng Technology Co., Ltd., Guangzhou, China.

Transparent medical PE tape (1.25 cm × 4.5 m) was acquired from Shenzhen Landemei Medical Equipment Co., Ltd., Shenzhen, China. Acrylic boxes (9.4 × 9.4 × 9.4 cm^3^) were purchased from Hubei Jingzhou Jingcheng United Trade, Jingzhou, China. Depilatory cream was acquired from Lijie Shi Company, London, UK. Imaging was performed with a Sony digital camera (ZV-1F, Tokyo, Japan). An AquaFlux AF200 device (Biox, London, UK) was utilized for the determination of TEWL. The skin composition structural analysis system (DUB—MSTC) and the skin polyguide physiological instrument were obtained from TPM of Germany. The Trichoscan HD dermatoscope was procured from Dermoscan, located in Regensburg, Germany. Guangzhou Vision Electronic Technology Co., Ltd., located in Guangzhou, China, provided the trichoscope (CBS-606). The laser speckle microcirculation imaging system (SQS-120P) was sourced from Shenzhen Shengqiang Technology Co., Ltd., Shenzhen, China. Other equipment, including the paraffin embedding device, paraffin slicing machine, and Leica orthostatic microscope, was manufactured by Leica in Wetzlar, Germany.

### 4.3. Modeling Groups

After a week of adaptation, the mice were anesthetized with 1% sodium pentobarbital. Once fully anesthetized, the fur from their cheeks to their forelimbs was removed using depilatory cream. The mice were adapted for 2 days. Subsequently, they were randomly assigned to one of three groups, with six mice in each group (Figure 1A).

Group I mice: In the control group, the skin extending from the left facial region to the corresponding upper limb was coated with PBS; the skin from the right portion of the face to the ipsilateral upper limb was the sensitive skin (SS) area. The right facial region was stripped with adhesive tape, followed by the application of 100 μL of propylene glycol and 6 μg/100 μL of capsaicin. The tape was peeled off once, and propylene glycol and capsaicin were applied daily for 6 days [15].

Group II mice: After tape exfoliation of the skin from the left and right cheeks to the ipsilateral upper limbs, 100 μL of propylene glycol, followed by 6 μg/100 μL of capsaicin, was applied daily on days 1–6 for sensitive skin modeling. Following this, the left cheeks of the mice were coated with 100 μL of 1% 4-TBLH for preventive application. The preventive application was initiated on day 1 and was continued for 7 consecutive days (SS+4-TBLH-Prevention). The right cheeks of the mice were coated with 100 μL of 1% 4-TBLH starting on day 4 for 4 consecutive days for a therapeutic application group (SS+4-TBLH-Therapy).

Group III mice: After tape stripping of the skin from the left and right cheeks to the ipsilateral upper limbs of the mice, 100 μL of propylene glycol, followed by 6 μg/100 μL of capsaicin, was applied daily on days 1–6 for sensitive skin modeling. Subsequently, a thin layer of 1% pimecrolimus ointment was applied to the left cheeks of the mice, and the ointment covered the cheek just enough that no closure was needed. The application started on day 1 and was continued for 7 consecutive days for the preventive application group (SS+Pimecrolimus-Prevention). The right cheeks of the mice were coated with a thin layer of 1% pimecrolimus ointment starting on day 4 for 4 consecutive days for the therapeutic application group (SS+Pimecrolimus-Therapy).

On day 7, data from various indices were collected for all groups separately, following the completion of the corresponding treatments.

### 4.4. Skin Erythema and Desquamation Reaction Evaluation Criteria

Skin responses (erythema, edema formation, and skin desquamation) were scored according to the adapted Draize and Kligman scales [20,21] (Table 5), and overall response intensity was classified according to the sum of the scores. The total score of 0–2 was classified as no reaction, 3–4 as mild reaction, 5–8 as moderate reaction, and >8 as strong reaction. Each group was scored and analyzed statistically.

### 4.5. Behavioral Testing for Itching and Tingling

On day 7, the mice were placed in an acrylic box for 15 min for environmental adaptation. Afterward, the left and right sides of the cheeks were treated according to their respective groups without anesthesia. Then, the mice were returned to the acrylic box, and two cameras were used to record the images from two positions for 45 min. Cheek scratching by the hind paws and cheek rubbing by the forelimbs of mice were observed as measures of itching and tingling sensations, respectively [37]. A scratching behavior was recorded when one or more rapid back-and-forth movements of the ipsilateral hind paw toward the cheek were recorded with the hind paw on the floor or the mouth. A rubbing behavior was recorded when the forelimb started at the back of the cheek and gently crossed from the caudal to the rostral side, with the medial arm of the forelimb playing a major role in this process. Scratching and wiping behaviors were counted based on image recordings.

### 4.6. Detection of TEWL and Epidermal Moisture Levels

After applying each substance and conducting behavioral imaging recordings for 45 min, the mice were transferred to the testing laboratory (laboratory temperature: 22 ± 1 °C, humidity: 50% ± 5%RH, and no wind (closed doors and windows, fans switched off)). All measurements are performed by the same operator. The mice received anesthesia through an intraperitoneal injection of 1% sodium pentobarbital and were kept quiet for 10 min using inhalation anesthesia before measurement. We selected a fixed time (10:00–11:00 am) and a fixed marker for the measurements.

The TEWL measurement instrument (AquaFlux AF200) was started 30 min in advance, the probe was linked, preheated to a steady state, and calibrated with the baseline using the built-in program. During the measurement process, the probe touched the skin vertically, the measurement time was ≥60 s until the reading was stable. The readings were repeated thrice with an interval of ≥1 min, and the average value was taken after the abnormal value was removed.

After skin hydration (DUB-MSTC) was started, a self-test was conducted to confirm that the capacitive sensor was responding properly. Next, we performed air (zero point) and standard plate calibration before each measurement using the device’s calibration plate. The probe was attached parallel to the skin to avoid sliding or tilting, and a single measurement time was 5 s. After reading the stable value, the same mark point was measured thrice with an interval of 10 s between readings, and the average value was taken as the result.

### 4.7. Detection of the Average Blood Perfusion of the Skin

When the mice were completely anesthetized, they were placed on the laser diffuse spot microcirculation imaging system detection stage. The detection height was adjusted, and the detection parameters were fixed when the system monitor indicated that the detection height was appropriate. Detection was then started, with three points detected on each side of the skin of each group of mice. Video recording of the average perfusion volume of the cutaneous microcirculation was conducted and recorded. Data were taken as the average value of the three points detected.

### 4.8. Trichoscopic Photos to Detect the Red Blood Cell Count Within the Skin

After the mice were anesthetized, the trichoscopic lens was directed at the skin of the mice, adjusting the light source and focal length to achieve optimal magnification (200×). Photographs were taken when the trichoscopic monitor displayed a clear field of view. More than three fields of each cheek skin of each group were photographed, and we recorded the number of visible veins in each photograph.

### 4.9. Lactic Acid Stimulation Test

After all testing indexes were completed, the mice were allowed to awaken fully. A 100 μL application of 0.5% lactic acid aqueous solution was applied to the skin on both cheeks of the mice in each group [15]. Behavioral image recordings were performed again for 45 min, and the scratching and wiping behaviors of the mice were statistically and analytically analyzed based on the behavioral image recordings. This analysis determined the index responsiveness of the sensitive skin model of the mice to the treatments of 4-TBLH and pimecrolimus. Independent samples *t*-tests were performed using SPSS.

### 4.10. Tissue Sampling and H&E Staining

On day 7, skin tissues from the modeling site of each group of mice were sampled separately. The collected skin tissues were rinsed with pre-cooled PBS on ice to remove blood, and the cleaned skin tissues were immersed in 4% paraformaldehyde for fixation. The tissues were then embedded in paraffin wax and sectioned. These sections were used for H&E staining according to standard protocols. Captured images were analyzed for skin thickness using Image J measurements.

### 4.11. Toluidine Blue Staining

A 1% aqueous solution of toluidine blue was prepared using toluidine blue powder. The skin tissue sections were stained using aqueous toluidine blue solution, and each section was photographed under a microscope with three 400× fields of view. The number of mast cells in each field of view was counted using Image J.

### 4.12. Immunohistochemical Staining

After routine dewaxing and hydration of paraffin sections, antigenic heat repair was performed using sodium citrate solution. Endogenous peroxidase activity was suppressed by the action of 3% H_2_O_2_ for 15 min and 10% goat serum BSA-blocking solution for 2 h. Primary antibodies (TNF-α: 1:250; TRPV1: 1:400; CD11b: 1:1000 and CD45: 1:1000) were incubated overnight at 4 °C. The sections were then incubated with a secondary antibody at room temperature for 1 h, developed using DAB for coloration, counterstained with hematoxylin, dehydrated through a gradient of ethanol, cleared by xylene, and sealed with resin. Images were captured using an orthogonal microscope. The positive expression of the samples (TNF-α and TRPV1) was detected by Image J software (V1.8) using the average optical density value (IOD), CD11b and CD45 immunohistochemical results were quantified by counting the number of positively expressing cells per unit area, and statistical analyses were performed [15].

### 4.13. Data Collection and Statistical Analysis

The measurement process was replicated on three separate occasions. Subsequently, statistical evaluation was carried out utilizing SPSS 26.0 software (IBM Corp., Armonk, NY, USA), and the data were presented in the format of mean ± standard deviation ($\bar{x}$ ± *s*). For comparisons between two groups, two independent samples *t*-tests were employed, whereas multiple comparisons were performed using one-way ANOVA with multiple comparisons. A difference was regarded as statistically significant when *p* < 0.05.

## 5. Conclusions

In this study, a mouse model of sensitive skin was treated with 4-TBLH (a TRPV1 antagonist) and 1% pimecrolimus ointment (a calmodulin phosphatase inhibitor). The model exhibited high sensitivity and responsiveness, as evidenced by significant treatment-dependent alterations in barrier function (desquamation, TEWL, and skin hydration), neurovascular responses (vascular dilation, itching/stinging behaviors), histological features (epidermal thickening), and molecular mechanisms (inflammatory cell infiltration, TNF-α, and TRPV1 expression). Notably, 4-TBLH and pimecrolimus demonstrated distinct regulatory effects depending on intervention timing (prophylactic vs. therapeutic). While 4-TBLH primarily alleviated neurogenic symptoms, pimecrolimus improved symptoms through immunomodulation, highlighting the model’s discriminative capacity in evaluating prevention and treatment strategies. Given its high responsiveness to diverse parameters, this model holds significant potential for future applications: rapid screening of novel efficacy ingredients, mechanistic exploration of neuro-immune-cutaneous barrier interactions, and serving as a standardized platform for translational studies on sensitive skin pathogenesis and treatment.

## Figures and Tables

**Table 1:** Total response score of each group (mean ± standard deviation).

Group	Average Score	Overall Skin Reaction Classification
Control	0.00 ± 0.00	No reaction/doubtful
SS	6.17 ± 0.75	Moderate reaction
SS+4-TBLH-Prevention	3.17 ± 0.41	Mild reaction
SS+4-TBLH-Therapy	4.33 ± 0.52	Mild reaction
SS+Pimecrolimus-Prevention	3.33 ± 0.52	Mild reaction
SS+Pimecrolimus-Therapy	4.50 ± 0.55	Mild reaction

Notes: *n* = 6, SS: sensitive skin; 4-TBLH: 4-tert-butylcyclohexanol.

**Table 2:** Changes in various skin physiological indices after treatment with 4-TBLH and pimecrolimus in the mice-sensitized skin model (mean ± standard deviation).

Group	PBS		SS+4-TBLH		SS+Pimecrolimus	
Indicators	Control	SS	Prevention	Therapy	Prevention	Therapy
TEWL	7.87 ± 1.87	18.35 ± 1.55	22.42 ± 2.67	28.40 ± 2.37	27.37 ± 3.16	30.44 ± 1.82
Hydration	55.83 ± 3.87	33.00 ± 1.90	38.83 ± 2.14	40.67 ± 1.51	30.67 ± 1.37	39.67 ± 1.37
Dilated capillaries	0.00 ± 0.00	20.67 ± 1.37	17.83 ± 1.72	19.17 ± 1.62	11.17 ± 1.72	12.00 ± 1.79
Perfusion volume	144.10 ± 0.69	154.70 ± 1.55	152.80 ± 1.37	151.30 ± 1.47	153.50 ± 1.73	152.50 ± 1.87
Itching	27.83 ± 2.56	81.67 ± 3.93	49.00 ± 2.68	56.83 ± 2.64	28.17 ± 2.48	13.17 ± 1.94
Tingling	21.83 ± 2.93	116.30 ± 5.20	67.50 ± 5.13	59.67 ± 5.75	26.33 ± 2.25	25.00 ± 1.87

Notes: *n* = 6, SS: sensitive skin; 4-TBLH: 4-tert-butylcyclohexanol.

**Table 3:** Changes in scratching and wiping behaviors before and after lactic acid test after treatment with 4-TBLH and pimecrolimus in a mouse sensitive skin model (mean ± standard deviation).

Group	PBS		SS+4-TBLH		SS+Pimecrolimus	
Indicators	Control	SS	Prevention	Therapy	Prevention	Therapy
Itching-before	31.67 ± 1.21	36.17 ± 2.48	32.17 ± 3.49	56.33 ± 2.94	41.17 ± 1.69	35.00 ± 1.79
Itching-after	36.17 ± 1.47	53.33 ± 4.23	50.33 ± 3.78	49.50 ± 2.88	26.67 ± 2.81	46.17 ± 3.31
Tingling-before	13.50 ± 1.38	22.17 ± 1.47	18.33 ± 0.82	28.00 ± 3.90	23.83 ± 2.71	35.00 ± 1.79
Tingling-after	16.67 ± 1.21	46.00 ± 1.41	33.33 ± 1.37	37.00 ± 2.37	21.00 ± 2.28	38.17 ± 2.93

Notes: *n* = 6, SS: sensitive skin; 4-TBLH: 4-tert-butylcyclohexanol.

**Table 4:** Changes in histological levels of the mouse sensitive skin model before and after treatment with 4-TBLH and pimecrolimus (mean ± standard deviation).

Group	PBS		SS+4-TBLH		SS+Pimecrolimus	
Indicators	Control	SS	Prevention	Therapy	Prevention	Therapy
Epidermal thickness/μm	9.62 ± 0.65	19.85 ± 1.68	29.02 ± 2.89	24.00 ± 2.22	24.39 ± 1.00	20.73 ± 1.85
Number of mast cells pr. mm^2^	8.50 ± 1.05	16.50 ± 1.87	23.67 ± 2.94	17.50 ± 1.05	19.67 ± 1.63	11.17 ± 1.72
Relative TNF-α IOD	7.75 ± 1.29	15.42 ± 2.04	21.81 ± 1.46	15.61 ± 2.09	15.01 ± 1.14	11.38 ± 0.78
Number of leukocytes pr.mm^2^	12.17 ± 2.04	42.83 ± 3.66	28.50 ± 2.74	30.00 ± 3.03	19.67 ± 2.73	28.83 ± 3.49
Number of T-cells pr.mm^2^	14.83 ± 3.77	47.67 ± 4.80	24.00 ± 2.37	27.67 ± 3.50	29.50 ± 3.62	19.67 ± 2.42
Relative TRPV1 IOD	0.36 ± 0.10	4.32 ± 0.61	4.13 ± 1.12	5.49 ± 0.74	1.96 ± 0.26	3.95 ± 0.99

Notes: *n* = 6, SS: sensitive skin; 4-TBLH: 4-tert-butylcyclohexanol.

**Table 5:** Skin reaction assessment criteria.

Score ^a^	Appearance of erythema
0	None
1	Very mild erythema
2	Well-defined erythema
3	Moderate erythema
4	Severe erythema
Score ^a^	Formation of edema
0	None
1	Very mild edema (almost perceptible)
2	Mild edema (defined area, beginning of swelling)
3	Moderate edema (swelling of ~1 mm)
4	Intense edema (growth > 1 mm and beyond application area)
Score ^b^	Skin desquamation
0	None
1	Dryness
2	Thin scales
3	Moderate scales
4	Large scales
Score	Overall skin reaction classification
0–2	No reaction/doubtful
3–4	Mild reaction
5–8	Moderate reaction
>8	Intense reaction

Note: ^a^: Draize scale; ^b^: Kligman scale.

## Data Availability

The additional data supporting the manuscript are available from the corresponding author upon request.

## Supplementary Materials

The following supporting information can be downloaded at https://www.mdpi.com/article/10.3390/ijms26094068/s1.

## Abbreviations

The following abbreviations are used in this manuscript:

TEWL	Transepidermal Water Loss
TRPV1	Transient receptor potential vanilloid 1
IL-6	Interleukin-6
VEGF	Vascular endothelial growth factor
4-TBLH	4-tert-butylcyclohexanol
KM	Kunming (mouse strain)
SS	Sensitive Skin
TS	Tape stripping
PG	1,2-Propylene Glycol
CS	Capsaicin
H&E	Hematoxylin and Eosin
TNF-α	Tumor Necrosis Factor-alpha
CD45	Cluster of Differentiation 45
CD11b	Cluster of Differentiation 11b
IOD	Integrated Optical Density
ANOVA	Analysis of Variance
BSA	Bovine Serum Albumin
DAB	3,3′-Diaminobenzidine
PBS	Phosphate Buffered Saline
SPF	Specific Pathogen-Free
