# Supplementary material for: Sensitivity of Various Indicators in a Mouse Sensitive Skin Model Treatment with 4-tert-Butylcyclohexanol and Pimecrolimus
Source: Int J Mol Sci. 2025 Apr 25;26(9):4068. doi: 10.3390/ijms26094068 (PMC12071757; doi:10.3390/ijms26094068)
Supplement: Supplementary file 1 [file ijms-26-04068-s001.zip › ijms-3551132-supplementary.pdf]

Supplementary data Figure S1

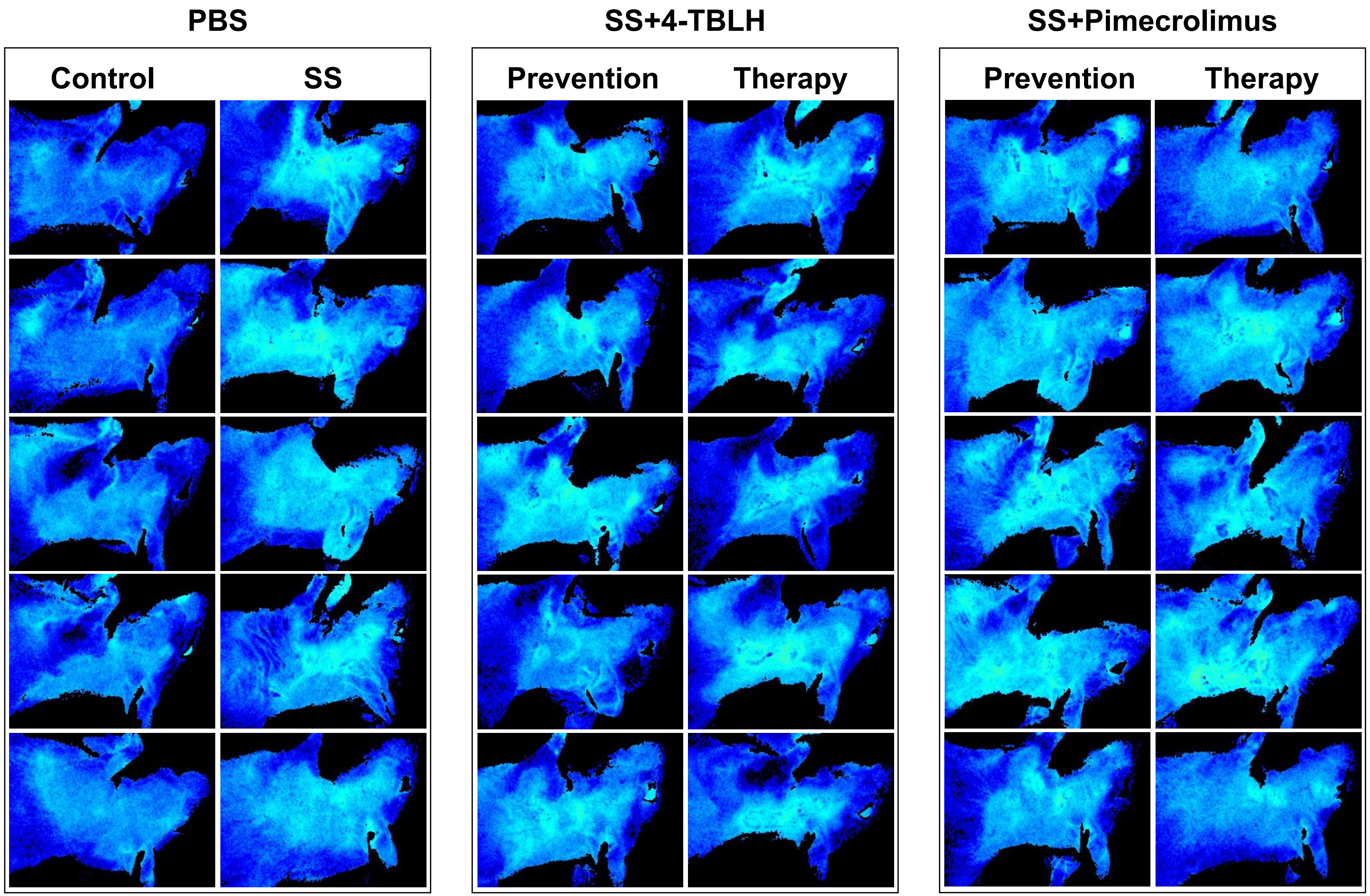

Supplementary Figure S1. Infrared blood flow imaging datasets for all experimental groups.
